# Structural Modeling of TRPA1 Ion Channel—Determination of the Binding Site for Antagonists

**DOI:** 10.3390/molecules27103077

**Published:** 2022-05-11

**Authors:** Alicja Gawalska, Marcin Kołaczkowski, Adam Bucki

**Affiliations:** Department of Medicinal Chemistry, Faculty of Pharmacy, Jagiellonian University Medical College, 30-688 Cracow, Poland; alicja.gawalska@doctoral.uj.edu.pl (A.G.); marcin.kolaczkowski@uj.edu.pl (M.K.)

**Keywords:** molecular modeling, TRPA1, TRPA1 antagonists, asthma, COPD, HC-030031

## Abstract

TRPA1 is a transmembrane cation channel, one of the most promising targets in the context of respiratory diseases. Its general structure has already been experimentally resolved, but the binding site of TRPA1 antagonists such as HC-030031, a model methylxanthine derivative, remains unknown. The present study aimed to determine the potential binding site of xanthine antagonists and to describe their binding mode, using a molecular modeling approach. This study represents the first attempt to bring together site-directed mutagenesis reports and the latest cryo-EM structure of an antagonist bound to TRPA1. Our research suggests that the core moiety of HC-030031 binds to a pocket formed by the TRP-like domain and the pre-S1, S4, S5 helices of one subunit. The structure, determined by cryo-EM, shows interactions of a core hypoxanthine moiety in the same area of the binding site, sharing the interaction of xanthine/hypoxanthine with Trp-711. Moreover, the predicted binding mode of HC-030031 assumes interaction with Asn-855, a residue demonstrated to be important for HC-030031 recognition in site-directed mutagenesis studies. Our model proved to be advantageous in a retrospective virtual screening benchmark; therefore, it will be useful in research on new TRPA1 antagonists among xanthine derivatives and their bioisosteres.

## 1. Introduction

The transient receptor potential (TRP) channel family is a group of transmembrane nonselective cation channels which includes seven subfamilies: canonical (TRPC), vanilloid (TRPV), melastatin (TRPM), mucolipin (TRPML), polycystin (TRPP), TRPN (NOMPC-like, no mechanoreceptor potential C), and ankyrin (TRPA1) [1]. All of the 28 distinct channels have been considered drug targets for multiple diseases such as chronic pain, inflammatory bowel disease, chronic obstructive pulmonary disease (COPD), and asthma [2,3]. Among them, TRPA1, the only member of the TRPA subfamily, is currently one of the most promising and studied targets in preclinical biological research, especially in the context of chronic and acute pain and respiratory diseases.

**Figure 1:**
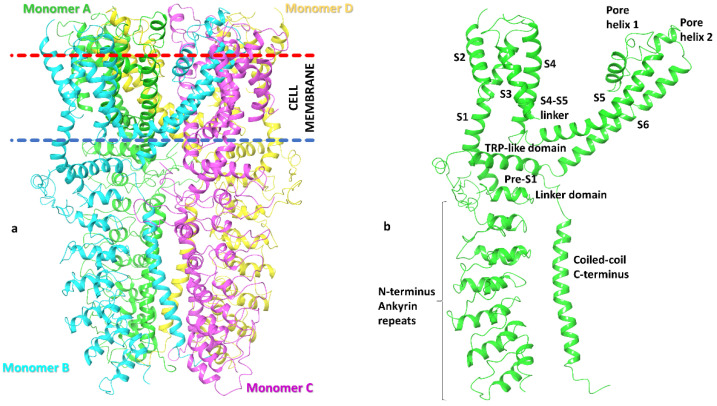
TRPA1 structural analysis: (**a**) distribution of the four homomonomers that form the TRPA1 ion channel and its arrangement in the cell membrane (based on information in OPM database); (**b**) structural details of a single TRPA1 subunit.

The TRPA1 ion channel is composed of four homomonomers forming a single pore, and is structurally characterized by an N-terminal fragment of 14–16 ankyrin repeats (30–34 amino-acid-long helix turn motifs), responsible for mediating protein–protein interactions with cytoskeletal proteins as well as the thermal and chemical sensitivity of the channel [4,5,6,7]. Each monomer contains six transmembrane domains (S1–S6), a reentrant pore loop in the transmembrane domain, and an intracellular coiled-coil structured C-terminal domain (Figure 1).

**Figure 2:**
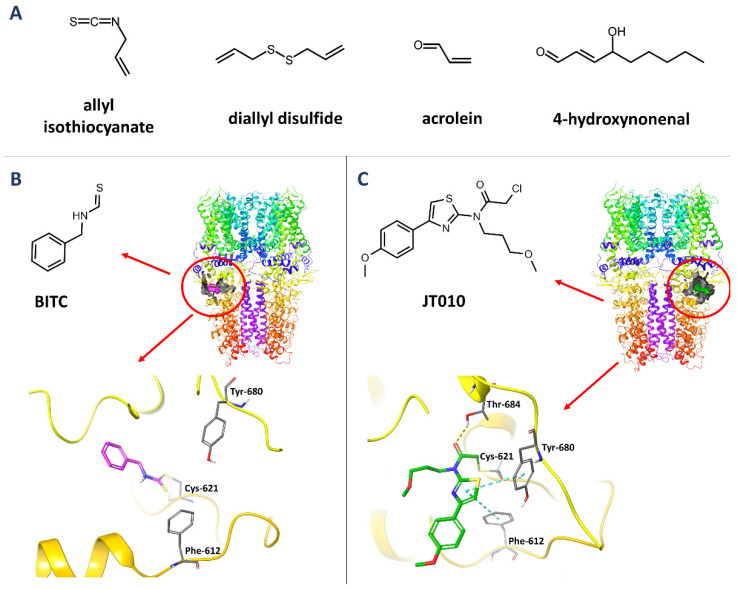
The structures of electrophilic TRPA1 agonists (**A**) and covalent agonists: BITC (**B**) and JT010 (**C**), their binding site location in the TRPA1 structure and their binding mode (yellow—hydrogen bonds, cyan—π-π stacking interactions) based on PDB: 6PQP and 6PQO [9], respectively.

TRPA1 is a ligand-gated ion channel that is modulated by voltage. It is activated in response to various endogenous and exogenous molecules. Among TRPA1 agonists, two groups can be distinguished: electrophilic and nonelectrophilic ligands. The electrophilic compounds, such as pungent natural products (allyl isothiocyanate, diallyl disulfide), environmental irritants (acrolein, vehicle exhaust components), and reactive metabolites (4-hydroxynonenal) (Figure 2A), activate the channel by covalently modifying cysteine residues. This applies primarily to Cys-621, but also to Cys-641, and Cys-665, all of which are located in the region comprising ankyrin repeats, linker, and pre-S1-helix that is responsible for allosteric regulation of TRPA1. Moreover, Lys-710, located in the pre-S1 helix, seems to play an equally important role, especially in the case of the binding of thiocyanates and ketoaldehydes. Furthermore, mass spectrometric analysis has shown that other lysine residues (Lys-704, Lys-787) are also crucial targets for electrophilic activation of hTRPA1 [8]. In 2020, Suo and co-authors presented cryogenic electron microscopy (cryo-EM) structures of TRPA1 in a complex with the covalent agonists JT010 and BITC, where they confirmed the location of the binding site between ankyrin repeats, pre-S1-helix, and the TRP-like domain as a coupling domain. Both compounds modify Cys-621 by reversible thiol-Michael adduct formation (BITC) and irreversible SN2 reaction (JT010). Additionally, the thiazole group of JT010 formed CH-π and sulfur-π interactions with Phe-612 and Tyr-680 (Figure 2B,C) [9]. The latest research conducted by Jianhua Zhao’s group indicates that electrophilic agonists act through a two-step process. The first phase is connected with Cys-621, which causes the reorientation of a cytoplasmatic loop to enhance nucleophilicity. Subsequently, in the second phase, Cys-665 is modified to stabilize the loop in an activated conformation [10].

**Figure 3:**
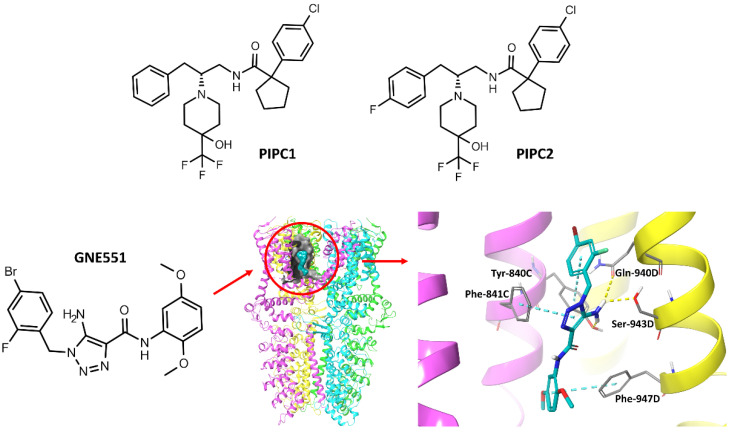
Structures of TRPA1 noncovalent agonists: PIPC1 and PIPC2 (piperidine carboxamide derivatives) and GNE551, showing its binding site location in the TRPA1 structure and its binding mode (yellow—hydrogen bonds, cyan—π-π stacking interactions) based on PDB: 6X2J [13].

The binding site of noncovalent agonists has not been directly described. However, the literature presents some assumptions about the hydrophobic pocket located at the interface of the pore helix-1, S5, and S6. It is worth noting that this binding site is similar to that of a known antagonist A-967079 [4]. Molecular modeling studies have indicated that piperidine carboxamides, a class of potent agonists described by Chernov-Rogan et al., and general anesthetics such as isoflurane, desflurane, and propofol interact right at that site. The lead compounds PIPC1 and PIPC2 were docked to homology models based on TRPV1/6, and interacted with Val-875, Ile-946, Ser-873, and Phe-909 (Figure 3) [11]. However, subsequent molecular modeling studies on the group of anesthetics suggested that isoflurane and propofol, while acting at the same binding site, have different binding modes focused on halogen bonds with Ser-873, Met-915, and Met-956 (isoflurane) or sulfur-aromatic interactions with Met-912 (propofol). Interactions with these amino acids are thought to be responsible for the agonism of volatile anesthetics at this target [12]. Both the above-mentioned predictions were confirmed by mutagenesis studies and appropriate functional assays. Recently, another binding site for a new noncovalent agonist GNE551 was discovered and confirmed with the cryo-EM structure of the TRPA1-GNE551 complex by Chen’s group [13]. The binding pocket was formed by the voltage-sensor-like domain (VSLD) of one subunit and the pore domain (S5–S6 helices) of the neighboring subunit and was characterized as a mostly hydrophobic pocket. Nevertheless, two hydrogen bonds with Tyr-840 and Ser-943 stabilized the complex, while Gln-940 was a critical residue for ion channel activation by GNE551 (Figure 3).

**Figure 4:**
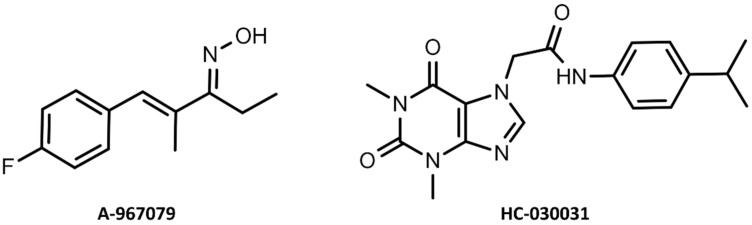
Structure of TRPA1 antagonists: A-967079 and HC-030031 (with a numbering scheme of 1,3-dimethylxanthine core).

In the treatment of pain and respiratory diseases, TRPA1 antagonists appear to be more pharmacologically relevant. A-967079 and HC-030031 are among the most widely studied TRPA1 antagonists discovered in recent years (Figure 4). The binding site of the former compound is located in the pocket formed by S5, S6, and the first pore helix. This site was first determined using a mutagenesis approach and then confirmed by a density map obtained through cryo-electron microscopy studies [1,4,10,11,14]. Paulsen et al. proposed that A-967079 forms hydrogen bonds with Ser-873 and/or Thr-874, both located at the bottom of the putative pocket, and π-π interactions with highly conserved Phe-909—a key residue, the mutation of which deprives A-967079 of its antagonistic effect [4]. This binding site for TRPA1 antagonists was confirmed in the experimental structure (PDB: 6WJ5), which represented the interaction pattern of the GDC-0334 inhibitor in the study by Lorena Riol-Blanco’s group [15]. Unfortunately, the binding site of HC-030031 could not be identified in these studies [4,16]. Moreover, it was proved that Phe-909 does not participate in the binding of the methylxanthine antagonist [12]. Further efforts have been made to find a binding site for HC-030031 and two main hypotheses have been published. The site-directed mutagenesis studies described in the paper by Gupta et al. indicated that interaction with a single amino acid residue Asn-855 is responsible for the inhibitory activity of HC-030031. The results of molecular dynamics (MD) simulation showed that HC-030031 formed a hydrogen bond with Asn-855 and the hydrophobic moiety of the molecule was located in a nearby hydrophobic pocket [17]. No other ligand–protein interactions were identified that would indicate the exact position of the compound at the binding site. On the other hand, Wenlei Ye and co-authors pointed out in their mutagenesis study that methylxanthine antagonists may bind in the pore in the vicinity of Asp-915, and its bioactive conformation is stabilized by the presence of acidic residues [18]. In addition, molecular modeling studies recently presented in an article by Kravchenko et al. described this region as a potential binding site for the TRPA1/TRPV1 antagonist HSV-DKH-0450 [19]. Given the lack of conclusive evidence, identification of a specific binding site and interactions formed by HC-030031 would be essential for the drug design of methylxanthine derivatives, which remain at the forefront of scientific interest. Considering the above, the aim of the present study was to determine the potential binding site of a representative of xanthine antagonists, compound HC-030031, and to illustrate its binding mode in order to support rational molecular design in this chemical space.

## 2. Results and Discussion

In the Sitemap program, 20 potential binding sites were identified and assessed (Table 1). The possible ligand binding sites were as follows: the region of the ankyrin repeats, the channel pore, the transmembrane domain (region around S1, S2, and S3), as well as the area between pre-S1, TRP-like domain, and S4–S5 helices, and below the TRP-like domain. The highest values of the druggability assessment score (DScore) were assigned to a place in the transmembrane region (1.213) for the 3J9P-based model, and to sites near the pre-S1/S4/S5/TRP-like domain (1.137) for the 6PQQ-based model.

The analysis of one subunit of the TRPA1 channel only by the Deepsite web server indicated three likely binding sites in the 3J9P-based model, which included the area around the linker domain, the transmembrane domain (S1/S2/S3), and the ankyrin repeats. In the case of the 6PQQ-based model homomonomer, only two binding sites were predicted. The favorably scored site was localized around the pre-S1/S4/S5/TRP-like domain which contains Asn-855, among others. The second binding site was indicated around the ankyrin repeats (Table 2).

As a result of TRPA1 structural analysis using the MetaPocket consensus program, three probable binding sites were predicted for each model (Table 3). Interestingly, for each of the models, two designated places were located near the pre-S1/S4/S5/TRP-like domain—the only difference was the individual subunits that constructed these areas. However, in the case of the TRPA1 channel, which is built of four homomonomers, the selected pocket would apply analogically to all subunits. The other two different sites were identified around the ankyrin repeats and the transmembrane domain for the 3J9P-based model and the 6PQQ-based model, respectively.

Castp3.0 determined 18 probable binding sites for both models, which are presented in detail in Table 4. In the case of the 3J9P-based model, the cavities were located mainly in the linker domain and ankyrin repeats, and, to a lesser extent, across the entire channel pore and in the transmembrane domain at the site formed by the helices S1, S2, and S3. On the other hand, the majority of sites in the 6PQQ-based model were found in the transmembrane area as well as in the entrance to the channel pore. Only a few of them were found at the intracellular channel pore. No binding sites were indicated near the pre-S1/S4/S5/TRP-like domain, contrary to the results from the previous algorithms.

It was observed that the potential binding sites were indicated across the entire TRPA1 structure, representing four main areas. The sites located within the ankyrin repeats and the transmembrane domain were similar to the already known binding sites of the TRPA1 ligands, particularly for the electrophilic agonists, which interact covalently with cysteine residues of the ankyrin repeats, but also for the nonelectrophilic agonists, and antagonists derived from A-967079 that bind in the region of the transmembrane domain. Due to the above-mentioned literature indications that HC-030031 does not interact with the TRPA1 ion channel in these areas [4,9,16], they were excluded from further analysis. The remaining putative binding sites were the actual channel pore, a fragment below the TRP-like domain and the space formed by the TRP-like domain and pre-S1, and the S4 and S5 transmembrane helices. However, the TRP-like domain and the region of the channel pore around Asp-915 already have established functions in ion channel activity. The TRP-like domain is involved in the activation of TRPA1 by electrophilic irritants and subserves allosteric regulation [4], and the channel pore in the vicinity of Asp-915 represents the site of TRPA1 pore constriction and is critical in controlling ion permeation [20]. The most prevalent binding sites were found in the pre-S1/S4/S5/TRP-like domain area (Table 5), and they were characterized by preferable values from the Sitemap (Table 1), Deepsite (Table 2), and MetaPocket2.0 (Table 3) scoring functions. Moreover, these active sites contained Asn-855 residue, which was found to be significant for the inhibitory effect of HC-030031 in research presented in the paper by Gupta et al. [17]. Therefore, this area was designated for a further step, which consisted of the docking of the TRPA1 antagonist HC-030031.

**Figure 5:**
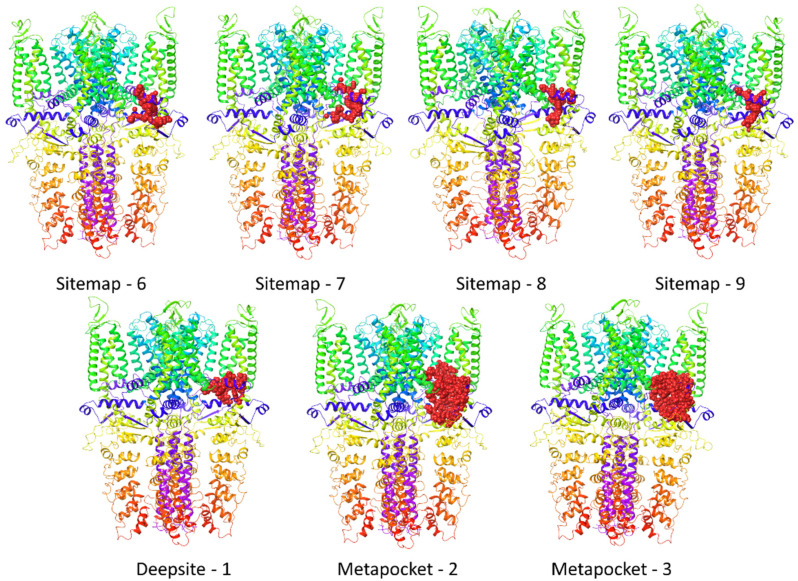
Selected binding sites for 6PQQ-based model from the group of pre-S1/S4/S5/TRP-like domain predictions.

In the search for potential binding sites, we used two TRPA1 ion channel models. For further steps of HC-030031 binding mode determination, starting with docking, we chose only one of them—the more reliable 6PQQ-based model. This model was prepared based on the recently released TRPA1 cryo-EM structure, which is characterized by a higher resolution and accuracy of matching atoms to the electron density map compared to 3J9P, while the predictors indicated analogous binding sites (among those regarded as relevant) for these two models (seven sites in the pre-S1/S4/S5/ TRP-like domain). The docking step was performed as an induced-fit docking procedure, which allows for conformational changes of the protein side chains and minor relaxation of the protein backbone that further enable the receptor to alter its binding site to be more compatible with the shape and functional group arrangement of the ligand. HC-030031 was docked to the TRPA1 ion channel at each of the seven potential sites that represented the selected group of pre-S1/S4/S5/TRP-like domain sites (Figure 5). Out of the docking results, one HC-030031-TRPA1 complex was selected for each site. That was the best rated position of the most common interaction pattern, characterized by favorable values of the scoring functions (IFDScore, docking score) and the presence of important (according to the literature) binding interactions (Table 6).

**Figure 6:**
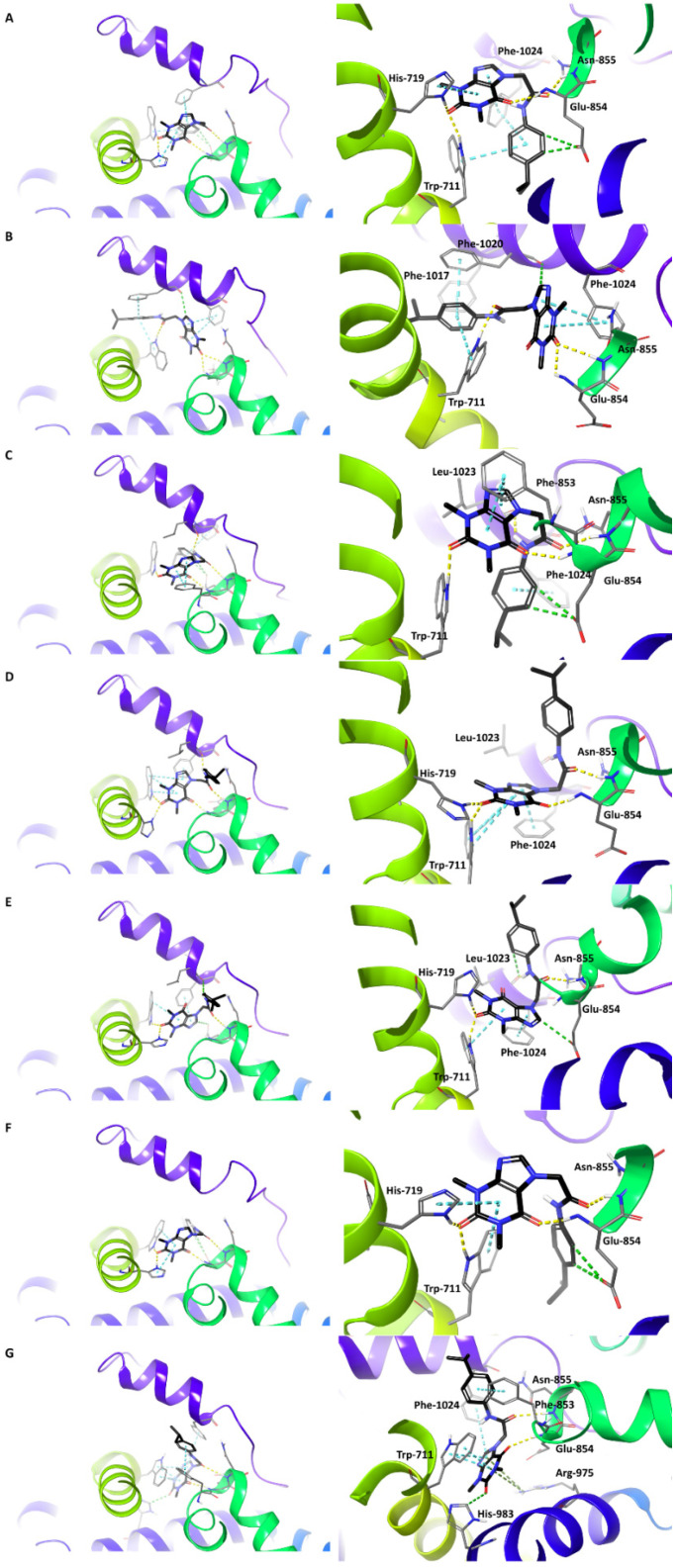
The predicted binding modes of HC-030031 for individual binding site models (**A**–**G**). View from the top of the channel (**left**) and the detailed illustration of the interactions (**right**), presented as follows: yellow—hydrogen bonds, green—aromatic hydrogen bonds, cyan—π-π stacking, dark green—cation-π interactions.

As depicted in Figure 6, HC-030031 was arranged in a similar manner at each of the designated binding sites. In every complex except B, the methylxanthine core was located in the space between the pre-S1 helix and S4–S5 linker, forming hydrogen bonds and/or π-π stackings with Trp-711. Additional stabilizing interactions were hydrogen bonds with Glu-854 and His-719, π-π stackings with Phe-1024 for the majority of complexes, and cation-π interactions with Arg-975 in the G complex. In all complexes, the methylxanthine core was perpendicular to the Trp-711 residue, excluding the G complex where it was located parallel to its plane. The substituent in position 7 of the ligand was arranged in two variants: it pointed “up” towards the transmembrane domain, which is represented by the D, E, and G complexes, and “down” towards the pre-S1 helix, which was illustrated by the remaining complexes. Interestingly, regardless of the variant, the carbonyl group of the amide bond formed a hydrogen bond with the main chain of Asn-855. In complex B only, the ligand’s substituent in position 7 was parallel to the helix formed by residues Leu-1016–Phe-1024, and a hydrogen bond was formed with Trp-711.

**Figure 7:**
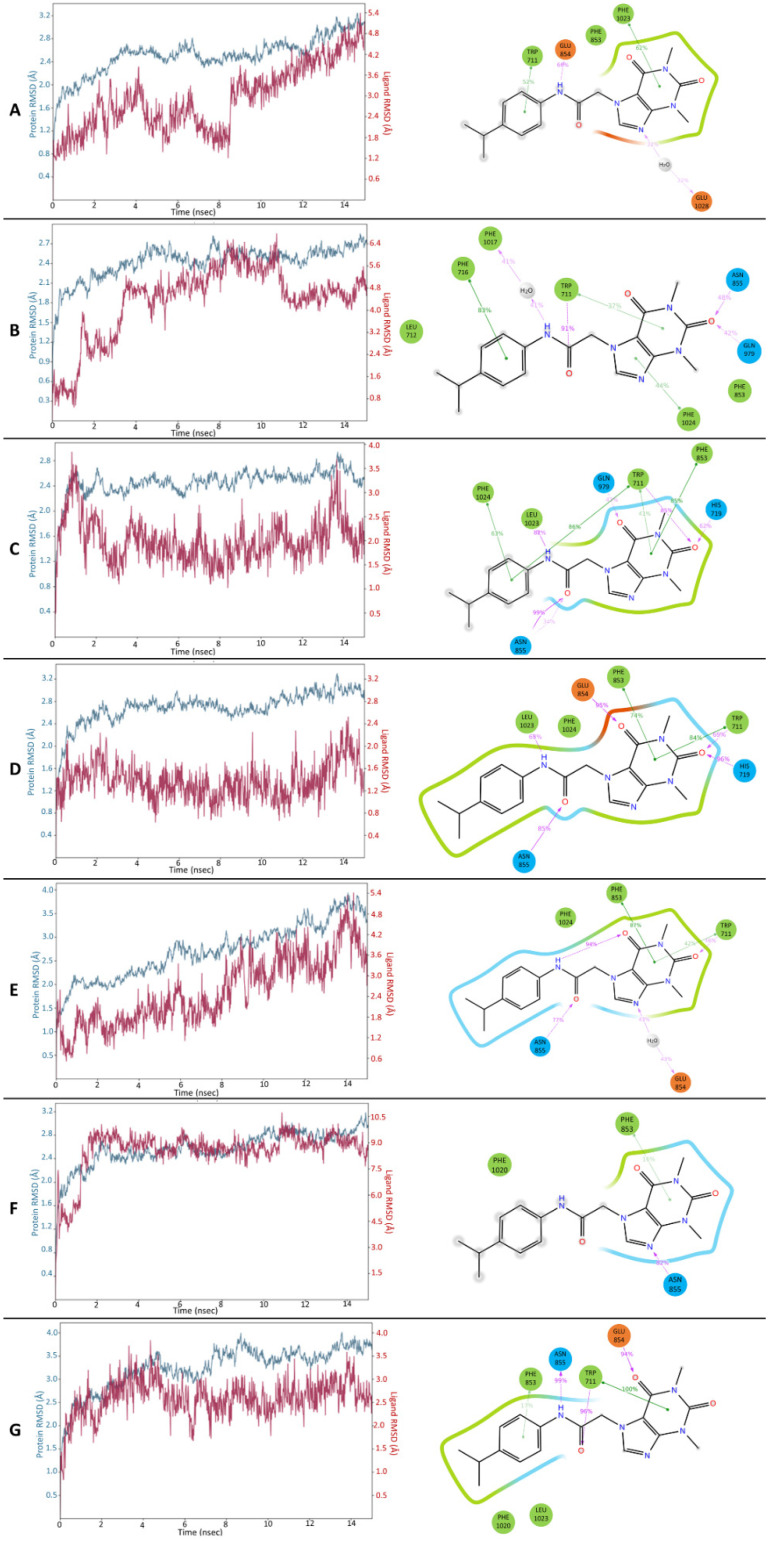
Results of 15 ns MD simulations for all complexes (**A**–**G**): RMSD plots (protein—blue, ligand—red), binding interactions formed, and their durability (percentage of the total simulation time).

The final complexes, collected in Figure 6, were subjected to MD simulations (15 ns) to assess the stability of the binding interactions. The C, D, and G complexes turned out to be the most stable over the entire simulation (Figure 7). The others showed large fluctuations in RMSD and no permanent conformations were established. Considering the stability of the binding interactions formed by the ligand with the protein, comparably persistent bonds occurred in the three complexes mentioned above. In the case of complex G, MD simulation results showed that after system relaxation, there was a change in the arrangement of the substituent in position 7 of HC-030031. Nevertheless, it H-bonded to the side chain of Asn-855 (which reflects the mutagenetic study results [17]), while maintaining the other characteristic interactions with Trp-711, Phe-853, etc. Moreover, this structure turned out to be the most stable one in MD (RMSD value below 4.0 Å); therefore, this representation of the HC-030031-TRPA1 structure was used for further research regarding complex G (Figure 7). The π-π stacking interaction of the xanthine pyrimidinedione ring with Trp-711 persisted for 100% of the simulation time, as did the hydrogen bonds with Asn-855, Glu-854, and Trp-711, which were observed for 99%, 94%, and 96% of the simulation time, respectively. The least established contact was the π-π stacking interaction with Phe-853 (17%). In complex D, hydrogen bonds with His-719, Glu-854, and Asn-855 were maintained for 96%, 95%, and 85% of the simulation time, respectively. The π-π stacking interactions with Trp-711 were observed in 84% of the simulation time. In complex C, the most stable interactions were hydrogen bonds with Asn-855, Trp-711, and Leu-1023 (99%, 85%, and 82% of the simulation time, respectively) as well as π-π stacking with Trp-711 and Phe-853 (86% and 85% of the simulation time). It was also characterized by high stability throughout the whole MD simulation in terms of RMSD (the protein 2.9 Å and the ligand 3.9 Å), similar to complex D (maximum RMSD of 3.2 Å for the protein and 2.5 Å for the ligand). During the simulations of the remaining complexes, ligand RMSD fluctuations up to 10.5 Å occurred. The above-described docking and MD results showed that complexes C, D, and G represent the most suitable binding modes of HC-030031. However, during the simulations of complexes C and G only, the substituent in position 7 of the ligand formed a permanent hydrogen bond with Asn-855 (99% of simulation time). Therefore, these two complexes were selected for further investigation—the protein structures served as models for docking and MD studies (referred to as models C and G).

**Figure 8:**
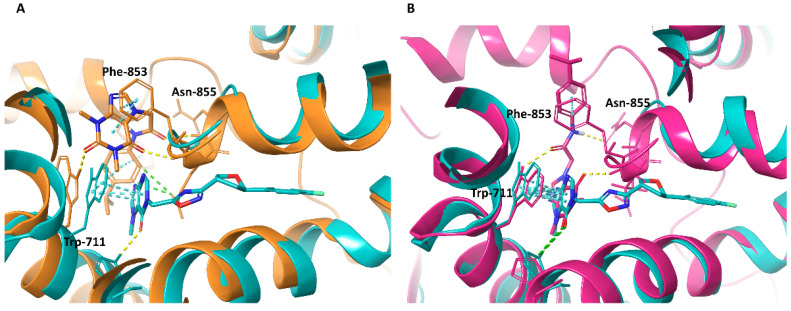
Binding modes of HC-030031 (predicted) and compound 21 (experimental) in the TRPA1 ion channel: (**A**)—complex C (orange) aligned with 7JUP (cyan); (**B**)—complex G (pink) aligned with 7JUP (cyan), with visible similarity of the binding interactions.

The comparison of the models developed as part of the present study with the cryo-EM structure of the TRPA1 ion channel bound to the hypoxanthine derivative compound 21 recently published by Terrett et al. (PDB ID: 7JUP, released 31 March 2021, after the described molecular modeling studies) revealed that in the selected binding site of complex G, the binding mode of the methylxanthine moiety was analogous to that of the hypoxanthine moiety from the experimental model (Figure 8B) [21]. In contrast, in the case of complex C, methylxanthine was located perpendicular to the hypoxanthine and above its site (Figure 8A). Both complexes differed substantially from the 7JUP representation in terms of the location of the large substituent—the amide moiety of HC-030031 was oriented upright, while the heterocyclic moiety of compound 21 was placed to the right (Figure 8A,B).

**Figure 9:**
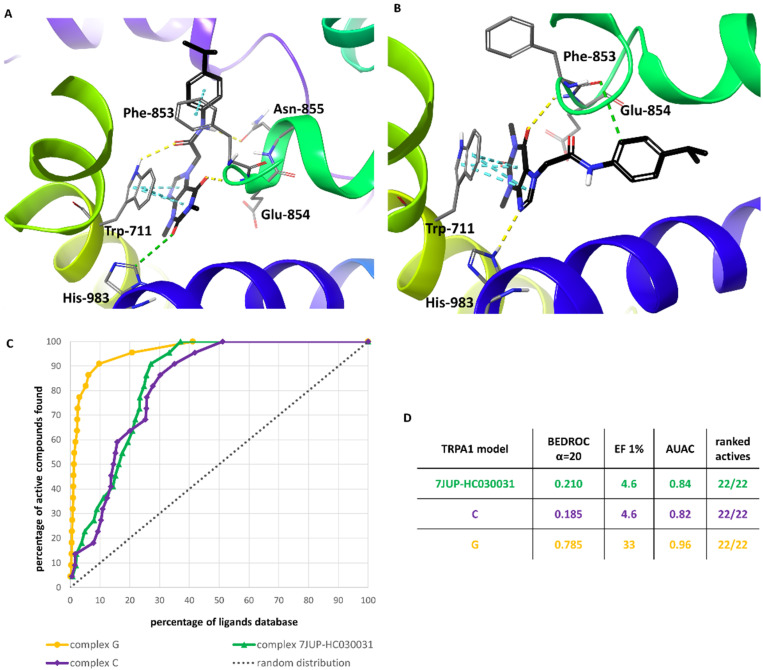
Binding modes of HC-030031 in complex G after relaxation protocol before MD simulation (**A**) and HC-030031 in the 7JUP-based model of the TRPA1 ion channel (**B**) The interactions are presented as follows: yellow—hydrogen bonds, green—aromatic hydrogen bonds, cyan—π-π stacking. The results of retrospective virtual screening (**C**)—enrichment plot for 7JUP-based model (green), model C (purple), and model G (orange) compared to the random distribution (dotted line); (**D**)—VS benchmark property summary).

In order to test the selected representations of the binding mode of methylxanthine antagonists in TRPA1, retrospective virtual screening studies were performed. The results (Figure 9C,D) showed that both models C and G recognized all 22 active ligands. Nonetheless, model G performed better than C in distinguishing the actives from the decoys, as indicated by the enrichment factor EF1% (33 vs. 4.6, respectively). In the 1% ligand database set (11.22 ligands), in the case of model G, nine active ligands were classified, whereas model C found only one active compound. The BEDROC parameter indicated the explicit usefulness of model G in virtual screening, as most of the active ligands were ranked before inactive decoys—BEDROC_α=20_ 0.785 (vs. 0.185 for model C). As shown in the enrichment plot, model G also had the largest area under the accumulation curve (AUAC = 0.96), which confirms that it can be considered to be the best representation of the antagonist binding site. The predicted binding mode of HC-030031 in model G, showing key interactions with Asn-855 and Trp711, is depicted in Figure 9A (for details, see Supplementary Materials: HC-030031-TRPA1_complex_G_relaxed.pdb file).

To compare the binding mode of HC-030031 itself in the newly available 7JUP TRPA1 conformational model with those found in the models developed as part of the present study, its structure was docked to a preprocessed 7JUP using Glide SP (Figure 9B). Moreover, the latter conformational model was tested in retrospective virtual screening under the same conditions as the previous models. The results showed that 7JUP, used as a model in VS, ranked all the active compounds, but only one of them was found in the first 1% of the compound library. This was reflected in the VS parameters, which were only slightly better than for model C, but distinctly lower than for model G (Figure 9C,D).

**Figure 10:**
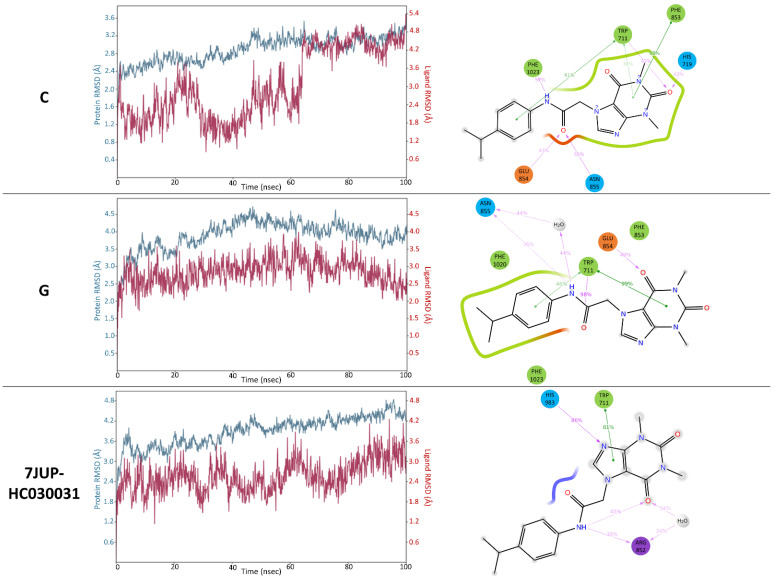
Results of 100 ns MD simulation for C, G, and 7JUP-HC-030031 complexes—RMSD plots (protein—blue, ligand—red), binding interactions formed, and their durability (percentage of the total simulation time).

To verify the stability of the predicted interactions and confirm the selection of the most probable representation of the binding mode of methylxanthine antagonist in TRPA1, additional 100 ns MD simulations were performed. The RMSD graphs show that complex G was characterized by the highest conformational stability of the ligand and fair stability of the ion channel (Figure 10). HC-030031 and the target protein in complex G established their conformations and were not subject to large RMSD fluctuations. The durability of the interactions was also favorable—the hydrogen bond and π-π stacking interactions with Trp-711 were observed for 98% and 99% of the simulation time. The hydrogen bond between the amide substituent in position 7 of the ligand and Asn-855 was also stable for over 80% of the simulation time, with the only difference being that it was mediated half the time by a water molecule. In the case of complex C, a hydrogen bond with Asn-855, as well as interactions with Trp-711, were established for shorter periods (55% and 31% of the simulation time), which confirmed the lowest ligand stability in the comparison. The results of the MD simulation of the 7JUP-HC-030031 complex took mid-range values. The RMSD fluctuations were slightly higher than for complex G, while the π-π stacking interactions with Trp-711 were less persistent (81% of the simulation time).

## 3. Materials and Methods

### 3.1. Preparation of TRPA1 Ion Channel Models

The complete protein preparation procedure was performed using modules of Small-Molecule Drug Discovery Suite (Schrödinger, LLC, New York, NY, USA, 2021). The models of the TRPA1 ion channel were based on its experimental representation in an inactive state, appropriate for modeling interactions of antagonists, derived from Protein Data Bank (codes: 3J9P and 6PQQ) [4,9]. The first closed-state ion channel structure (PDB: 3J9P) was published in 2015 as a result of an experiment using cryo-electron microscopy, determined in the presence of the abovementioned potent antagonist A-967079. A relatively low resolution of 4.24 Å might indicate an inaccurate electron potential density map and difficulties in matching the atomic structure. The consequence of this is the lack of certain protein fragments (63% of the structure): the N-terminus (1–445) and C-terminus amino acids (1079–1119), as well as undefined loops in the final structure (664–679, 748–763, 786–802) [22]. Another experimental structure of the TRPA1 ion channel (PDB: 6PQQ) was released in 2020 and represents the C621S mutation, which forces its inactive conformation. It was resolved using cryo-electron microscopy as well, but with a much better resolution of 2.81 Å. Its representation was also lacking fragments, but only 48% of the structure: the N-terminus (1–445), C-terminus (1079–1119), and several loops (669–676, 754–760, 1026–1038) [23]. The structures were then prepared using Protein Preparation Wizard and missing amino acid side chains were added. Missing loops were built using a homology modeling procedure with Prime Structure Prediction Wizard (Table 7). This process was based on the TRPA1 sequence derived from the UniProt database (entry O75762). Finally, the refinement of the added loops was made with an implicit membrane, which was set according to data obtained from the OPM database (https://opm.phar.umich.edu, accessed on 26 February 2021) [24].

### 3.2. TRPA1 Binding Site Prediction

Potential binding sites in the TRPA1 ion channel were explored and characterized in both the prepared models using the following programs and web services: SiteMap, DeepSite, MetaPocket, and Castp3.0. The methodologies of these tools are based on various types of algorithms. Using multiple approaches was expected to yield reliable results for possible binding sites in the TRPA1 ion channel. The obtained results were grouped according to the protein regions where the sites were found, and then the structures were analyzed based on the scoring function value.

#### 3.2.1. SiteMap (Schrödinger, LLC, New York, NY, 2021)

SiteMap s an energy-based cavity-finding algorithm which identifies probable binding sites through detection, characterization, and evaluation of cavities [25,26]. The scoring function is the DScore, and a value higher than 1 suggests a druggable site. The SiteMap protein binding site evaluation was carried out using the default settings (at least 15 site points were required per each of maximum 20 reported sites; the less restrictive definitions of hydrophobicity and standard grid were set).

#### 3.2.2. MetaPocket 2.0 Server

MetaPocket2.0 (https://projects.biotec.tu-dresden.de/metapocket/index.php, accessed on 19 June 2020) is a meta server used to identify ligand binding sites on a protein surface, which combines four approaches of site prediction: grid-based LIGSITE, two spheres-based PASS, SURFNET, and energy-based Q-SiteFinder, which improves the prediction success rate [27]. The batch files for the calculations on this webserver were the .pdb files of the prepared TRPA1 structure models. The settings provided allowed up to 10 potential binding sites to be detected.

#### 3.2.3. DeepSite

DeepSite ((https://playmolecule.org/deepsite/, accessed on 29 June 2020) is a predictor of protein binding pockets based on deep neural networks [28]. For calculations, both the prepared TRPA1 models were uploaded and chain (homomonomer) A was selected, due to the limitation of the maximum size of the system to 1000 amino acids. The scoring function ranges from 0 (not-pocket) to 1 (definitely-a-pocket).

#### 3.2.4. CASTp 3.0

CASTp 3.0 (http://sts.bioe.uic.edu/castp/index.html?4jii, accessed on 13 July 2020) is a web server focused on alpha-shape analysis capable of locating, defining, and measuring geometric and topological properties of protein structures as well as improving visualization of protein pockets [29,30]. Calculations were performed separately for each of the prepared models, using default settings (radius probe = 1.4 Å).

### 3.3. The Procedure of HC-030031 Docking to TRPA1 Binding Sites

Docking of HC-030031 through the induced-fit docking procedure was performed at each of the designated sites. The cell membrane was included in the structure of the TRPA1 ion channel. The docking process was carried out with the default settings; no constraints were set. The obtained results were analyzed in terms of the scoring function values (IFDScore and docking score), as well as the presence of binding interactions, favorable/unwilling contacts/clashes, and the repeatability of a given position. The best-ranked complex was selected for each binding site.

### 3.4. MD Simulations of HC-030031-TRPA1 Complex

The selected TRPA1-HC-030031 complexes were subjected to a 15 ns MD simulation and thereafter, the two most stable complexes were subjected to an additional 100 ns simulation. All MD simulations were performed using the Desmond GPU package. Systems for the simulations were prepared using the System Builder module. TRPA1-HC-030031 complexes obtained in the IFD process were placed in an orthorhombic cell, with size adjusted to a minimum and the SPC solvent model set. The POPC membrane was added to the system and an appropriate number of counter ions to maintain charge neutrality were added. After initial model relaxation, 15 ns and 100 ns simulations utilizing the OPLS_2005 force field were run in NPγT ensemble, and trajectories were saved in 15 and 100 ps intervals, respectively. Simulation interaction protocols were generated to calculate RMSD plots and interaction diagrams.

### 3.5. Validation of the Selected HC-030031-TRPA1 Interaction Models—The Retrospective Virtual Screening Benchmark

The most stable HC-030031-TRPA1 complexes were benchmarked in retrospective virtual screening using 22 high-affinity ligands and 1100 decoys. The group of active ligands, composed of methylxanthine derivatives and its bioisosteres, was derived from the patent review by Preti et al. [31] (list of individual ligand structures in Supporting Information Table S5). Based on their structures, 1100 property-matched decoys were generated using DUD-E server [32]. All the compounds were optimized with Ligprep using the following settings: retain specified chiralities and generate at most 1 per ligand. The procedure resulted in a total of 1671 tautomers and optical isomers of the decoy structures. Ionization of the active compounds was predicted in pH 7 ± 2. The input ionization of decoys was retained, since DUD-E generates proper structures. Grids containing hydrogen bond constraints on Asn-855 were prepared based on the selected complexes—the structures resulting from the relaxation process preceding the MD simulations. Ligands were docked using the Glide SP procedure, generating one final position. The results were analyzed using parameters determined using Enrichment Calculator: BEDROC (Boltzmann-Enhanced Discrimination of Receiver-Operating Characteristic), EF1% (Enrichment Factor), and AUAC (Area under the accumulation curve) [33,34]. The BEDROC parameter measures the early recognition of actives from the database and a value of α = 20.0 corresponds to 80% of the total score being accounted for the top 8% of the database [35]. BEDROC takes the values 0–1, where 1 means that all active molecules are classified before inactive compounds at the beginning of the screening results [33]. The enrichment factor (EF) was calculated for the top 1% of the database (EF1%). It characterizes the increase of the number of active compounds found in a given set ranked according to the value of the evaluating function, in relation to random distribution [34]. Finally, AUAC was used as a function of the average rank of actives and summary estimate of test performance, taking values from 0 (useless test) to 1.0 (ideal screen performance) [33].

### 3.6. Comparison of the Optimized Models with the PDB Structure 7JUP

The structure of TRPA1 (PDB ID 7JUP) bound with an antagonist, namely a hypoxanthine derivative (1-({3-[(3R,5R)-5-(4-fluorophenyl)oxolan-3-yl]-1,2,4-oxadiazol-5-yl}methyl)-7- methyl-6,7-dihydro-1H-purin-6-one; compound 21) was processed using Protein Preparation Wizard [21]. The selected C and G complexes were aligned with the experimental structure using Protein Structure Alignment to compare the arrangement and binding interactions of HC-030031 with the model antagonist. The 7JUP protein structure then served as a grid for HC-030031 docking in the Glide SP procedure. The molecule was bound in a similar way as compound 21—hydrogen bond with His-983 and π-π stacking with Trp-711. The protein structure was therefore considered to be an alternative conformational model of interactions for HC-030031 and was verified in a retrospective virtual screening according to the process described in Section 3.5, the only difference being constraints set to His-983. The selected 7JUP-HC-030031 complex was subjected to a 100 ns MD simulation, carried out following the process from Section 3.4.

## 4. Conclusions

Our research showed that the most likely binding mode of xanthine antagonists represented by HC-030031 in the TRPA1 ion channel was reflected by complex G, where the ligand interacted in a pocket formed by the TRP-like domain and the pre-S1, S4, and S5 helices of one subunit. HC-030031 established two crucial interactions: hydrogen bonding with Asn-855 and π-π stacking with Trp-711. The selected binding pattern confirms the experimental studies described in the article by Gupta et al., indicating the importance of the interaction with Asn-855 for the antagonistic activity of HC-030031 [17]. Moreover, the general arrangement of the xanthine core in the hydrophilic part and the substituent in the hydrophobic part of the active site are consistent with the results published in this paper. Furthermore, the results are in line with the studies by Terrett et al., since the arrangement of the methylxanthine core in the developed complex and its interaction with Trp-711 are analogous to the ones of hypoxanthine in the PDB 7JUP complex [21]. A new insight reported here is the π-π stacking interaction of HC-030031 with Trp-711, which might play an equally important role in the binding of other xanthine derivatives in the TRPA1 ion channel. This study represents the first attempt to determine the binding site and describe the interactions of HC-030031, an approach that brings together experimental reports on site-directed mutagenesis and the latest cryo-EM structure of the other antagonist bound to TRPA1. The results prove the usefulness of the molecular modeling methodology in the identification of binding sites of druggable targets, explaining the interactions with their ligands. Thus, this paper paves the way for further studies (e.g., a prospective virtual screening) in search of new potential TRPA1 antagonists in the group of xanthine derivatives as well as their bioisosteres, and hence potential new drugs for the treatment of neuropathic pain, asthma, and COPD.

## Figures and Tables

**Table 1:** Sitemap binding site prediction results. The number of sites provided in the presented areas of models based on 3J9P and 6PQQ, and their average Dscore values (the higher the better). Total average Dscore is shown for all selected sites in a given area for both models.

		Ankyrin Repeats 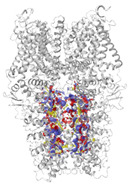	Channel Pore 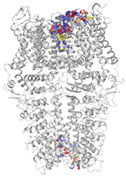	Transmembrane Region 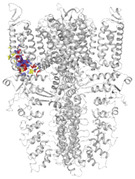	Pre-S1/S4/S5/ TRP-Like Domain 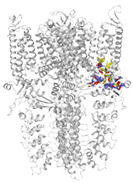	Below TRP-Like Domain Area 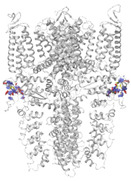
**3J9P-based model**	number of sites	6	3	1	5	5
	average Dscore ± SD	1.029 ± 0.080	1.064 ± 0.110	1.213	1.019 ± 0.027	1.045 ± 0.038
**6PQQ-based model**	number of sites	4	4	1	4	6
	average Dscore ± SD	1.011 ± 0.014	1.010 ± 0.052	1.041	1.137 ± 0.047	0.971 ± 0.056
**Total average Dscore ± SD**		1.020 ± 0.013	1.037 ± 0.038	1.127 ± 0.122	1.078 ± 0.074	1.008 ± 0.052

**Table 2:** Web server Deepsite binding site prediction results for models based on 3J9P and 6PQQ: graphical illustration and evaluation score (the higher the better).

		Binding Site 1	Binding Site 2	Binding Site 3
**3J9P-based model**	score	0.9989	0.9968	0.9991
	site	linker domain	transmembrane domain (S1/S2/S3)	ankyrin repeats
		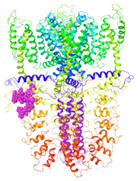	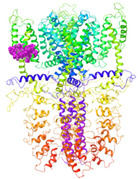	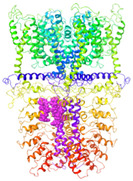
**6PQQ-based model**	score	0.9997	0.9987	
	site	pre-S1/S4/S5/TRP-like domain	ankyrin repeats	
		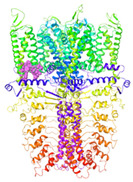	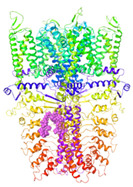	

**Table 3:** MetaPocket2.0 binding site prediction results for models based on 3J9P and 6PQQ: graphical illustration and evaluation score.

		**Binding Site 1**	**Binding Site 2**	**Binding Site 3**
**3J9P-based model**	score	4.39	3.76	3.70
	Site	pre-S1/S4/S5/TRP-like domain	Ankyrin repeats	pre-S1/S4/S5/TRP-like domain
		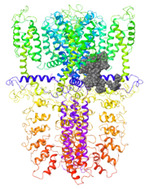	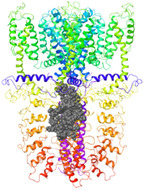	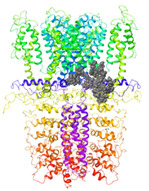
**6PQQ-based model**	score	8.34	5.21	4.52
	Site	Channel pore	pre-S1/S4/S5/TRP-like domain	pre-S1/S4/S5/TRP-like domain
		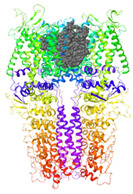	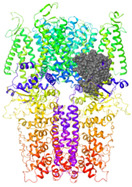	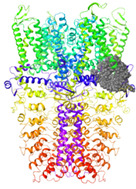

**Table 4:** Prediction of binding sites from Castp3.0 for models based on 3J9P and 6PQQ: number of sites and graphical illustration (red—the predicted sites).

		Ankyrin Repeats	Channel Pore	Transmembrane Region	Below TRP-Like Domain Area
**3J9P-based model**	number of sites	8	3	3	4
	sites	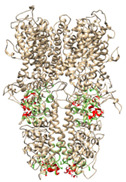	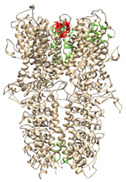	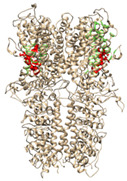	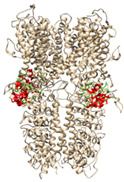
**6PQQ-based model**	number of sites	5	2	11	
	sites	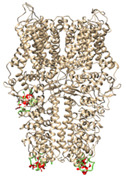	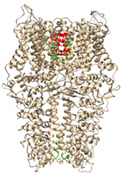	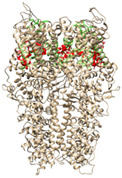	

**Table 5:** Summary of all predicted binding sites for models based on 3J9P and 6PQQ. Sites excluded from further investigation are marked in red. The most prevalent binding site, consistent with experimental reports, is marked in green.

	3J9P-Based Model	6PQQ-Based Model	Total
Ankyrin repeats	11	6	17
Channel pore	4	6	10
Transmembrane region	3	3	6
**pre-S1/S4/S5/ TRP-like** **domain^18^**	7	7	14
TRP-like domain	5	6	11

**Table 6:** Summary of HC-030031 induced-fit docking (IFD) results to individual binding sites: IFDScore and Docking score.

HC-030031-TRPA1 Complex	Binding Site	IFDScore (kcal/mol)	Docking Score (kcal/mol)
**A**	Sitemap-6	−4983.80	−9908
**B**	Sitemap-7	−4982.47	−9681
**C**	Sitemap-8	−4985.76	−9874
**D**	Sitemap-9	−4990.55	−12,833
**E**	Metapocket-2	−4986.84	−11,574
**F**	Metapocket-3	−4981.45	−9303
**G**	Deepsite-1	−4984.21	−10,698

**Table 7:** The prepared models of the TRPA1 ion channel: original structures, amino acids in the cell membrane, and added loops in the process of homology modeling.

	3J9P-Based Model	6PQQ-Based Model
**PDB original structure**	3J9P	6PQQ
**Membrane amino acids**	720–739	719–738
	767–785	768–793
	807–823	799–820
	830–850	833–853
	870–892	863–891
	934–957	935–961
**Added loops in each homomonomer**	664–669	669–676
	748–763	754–760
	786–802	1026–1038

## Data Availability

The initial TRPA1 structures were downloaded from PDB database: 3J9P (https://www.rcsb.org/structure/3J9P, accessed on 17 February 2020), 6PQQ (https://www.rcsb.org/structure/6PQQ, accessed on 17 February 2020) and 7JUP (https://www.rcsb.org/structure/7JUP, accessed on 1 April 2021); TRPA1 sequence was taken from the UNIPROT database (https://www.uniprot.org/uniprot/O75762, accessed on 16 February 2020); The complete molecular modeling procedure was performed using modules of Small-Molecule Drug Discovery Suite (Schrödinger Inc., v11.15.0, Schrödinger, LLC, New York, NY, 2021) (https://www.schrodinger.com/, accessed on 17 February 2020); the other data presented in this work are available in the article and Supplementary Materials.

## Supplementary Materials

The following supporting information can be downloaded at: https://www.mdpi.com/article/10.3390/molecules27103077/s1, Table S1: SiteMap TRPA1 binding site prediction results; Table S2: Deepsite TRPA1 binding site prediction results; Table S3: Metapocket 2.0 TRPA1 binding site prediction results; Table S4: Castp3.0 TRPA1 binding site prediction results; Table S5: List of active ligands used in retrospective virtual screening; HC-030031-TRPA1_complex_G_relaxed (.pdb file).

## Sample Availability

Samples of the compounds are not available from the authors.
