# Supplementary material for: Structural Modeling of TRPA1 Ion Channel—Determination of the Binding Site for Antagonists
Source: Molecules. 2022 May 11;27(10):3077. doi: 10.3390/molecules27103077 (PMC9145427; doi:10.3390/molecules27103077)
Supplement: Supplementary file 1 [file molecules-27-03077-s001.zip › Gawalska et al. Supplementary Materials Table S1-S5.pdf]

Supplementary Materials

Structural modeling of TRPA1 ion channel – determination of the binding site for antagonists

Alicja Gawalska, Marcin Kołaczkowski and Adam Bucki *

Jagiellonian University Medical College, Faculty of Pharmacy, Department of Medicinal Chemistry, 30-688 Cracow, Medyczna 9, Poland
* Correspondence: adam.bucki@uj.edu.pl

Table S1: SiteMap TRPA1 binding site prediction results
Table S2: Deepsite TRPA1 binding site prediction results
Table S3: Metapocket 2.0 TRPA1 binding site prediction results
Table S4: Castp3.0 TRPA1 binding site prediction results
Table S5: List of active ligands used in retrospective virtual screening.

Table S1: SiteMap TRPA1 binding site prediction results

		Ankyrin repeats<br>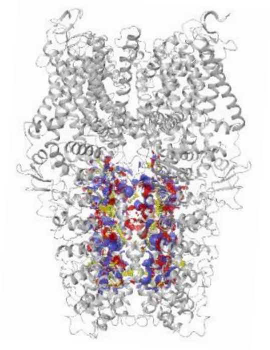	channel gap<br>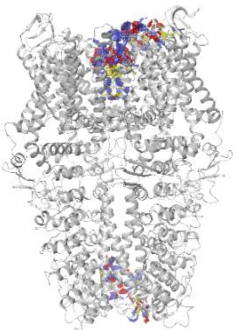	Transmembrane region<br>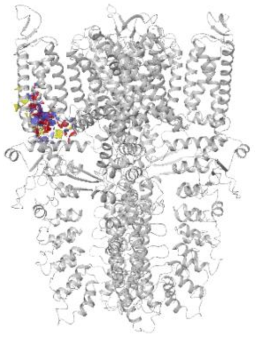	pre-S1 / S4 / S5 / TRP-like domain<br>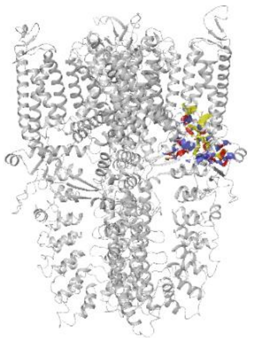	below TRP-like domain area<br>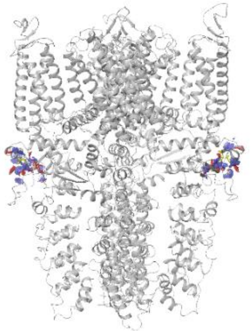
3J9P-based model	number of sites	6	3	1	5	5
	DScore - site	0.955 - s1<br>1.013 - s4<br>1.026 - s7<br>1.184 - s15<br>1.000 - s17<br>0.994 - s18	1.017 - s3<br>0.985 - s10<br>1.189 - s19	1.213 - s20	1.030 - s2<br>1.010 - s5<br>1.024 - s6<br>0.980 - s13<br>1.054 - s14	1.052 - s8<br>1.064 - s9<br>1.065 - s11<br>1.068 - s12<br>0.977 - s16
	average score	1.029	1.064	1.213	1.019	1.045
6PQQ-based model	number of sites	4	4	1	4	6
	DScore - site	1.023 - s1<br>1.016 - s2<br>1.015 - s3<br>0.991 - s5	1.075 - s4<br>1.029 - s13<br>0.961 - s15<br>0.976 - s17	1.041 - s19	1.089 - s6<br>1.118 - s7<br>1.172 - s8<br>1.172 - s9	0.989 - s12<br>0.958 - s14<br>0.932 - s16<br>0.929 - s18<br>0.939 - s20<br>1.076 - s12
	average score	1.011	1.010	1.041	1.137	0.971

Table S2: Deepsite TRPA1 binding site prediction results

		Binding site 1		Binding site 2		Binding site 3	
3J9P-based model	score	0.9988941550254822		0.996812641620636		0.9990683197975159	
	site	linker domain		transmembrane domain (S1 / S2 / S3)		ankyrin repeats	
		Tyr-655	Asn-619	Asn-724	Tyr-812	Tyr-629	Leu-1051
		Ile-656	Cys-608	Tyr-726	Ser-780	Lys-593	Arg-1050
		Glu-657	Leu-609	Tyr-849	Ser-781	Ile-627	Tyr-1049
		Tyr-658	Lys-610	Gly-729	Ile-782	Gln-594	Lys-1048
		Asn-659	Ile-611	Tyr-842	Phe-783	Arg-592	Glu-1047
		Phe-660	Lys-620	Asn-845	Gly-784	Lys-591	Lys-1044
		Leu-686	Cys-621	Glu-808	Tyr-785	Asn-999	Leu-1045
		Tyr-680	Gly-618	Tyr-809		Asp-1053	Met-1042
		Ala-685	Glu-607			Lys-1052	
6PQQ-based model	score	0.9997281432151794		0.9987033605575562			
	site	pre-S1 / S4 / S5 / TRP-like domain		ankyrin repeats			
		Arg-852	Gln-1028	Lys-591	Lys-1052		
		Met-720	Asn-855	Asn-590	Leu-1051		
		His-719	Gln-854	Arg-592	Tyr-1049		
		Phe-716	Cys-856	Arg-557	Lys-1048		
		Trp-711	Phe-853	Gln-558	Lys-1046		
		Phe-1020	Gln-979	Leu-1054	Leu-1045		
		Leu-1023	Phe-1024	Asp-1053			

Table S3: Metapocket 2.0 TRPA1 binding site prediction results

		Binding site 1			Binding site 2			Binding site 3		
3J9P-based model	score	4.39			3.76			3.70		
	Site	pre-S1 / S4 / S5 / TRP-like domain			Ankyrin repeats			pre-S1 / S4 / S5 / TRP-like domain		
		Ile-858B	Ser-873C	Glu-705B	Ile-611C	Asp-606C	Leu-683C	Glu-705C	Gln-1031C	Asn-855C
		Met-862B	Asn-855B	Arg-975B	Gly-618C	Leu-584C	Leu-588C	Glu-1032C	Pro-1034C	Arg-872D
		Val-967B	Cys-856B	Glu-854B	Lys-620C	Ile-633C	Leu-552C	Ile-1033C	Ile-695C	Ile-858C
		Gln-968B	Val-875C	Ser-972B	Tyr-658C	Thr-634C	Ala-556C	Asn-699C	Lys-969C	Gln-768C
		Ala-971B	Ile-860B	Asp-1037B	Leu-609C	Leu-640C	Val-564C	Pro-701C	His-970C	Cys-856C
		Thr-869C	Lys-868C	Ile-695B	Asn-619C	Pro-631C	Phe-583C	His-700C	Ala-971C	Val-865D
		Leu-959C	Pro-1034B	Asn-699B	Cys-608C	Met-634C	Val-595C	Val-1005C	Asp-1037C	Asp-966D
		Ile-964B	Gln-1031B	His-970B	Glu-607C	Ile-600C	Leu-568C	Arg -652C	Ser-972C	Val-967D
		Phe-859B	Glu-1032B	Leu-973B	Tyr-654C	Ile-599C	Thr-598C	Tyr-706C	Leu-973C	Lys-974D
		Leu-955C	Gly-958C	Lys-969B	Ile-656C	Lys-635C	Cys-621C	Val-702C	Arg-975C	Asp-963D
		Val-865C	Leu-871C	Met-709B	Glu-657C	Ala-685C	Ala-688C	Met-709C	Glu-854C	
		Arg-872C	Ile-1033B	Tyr-706B	Asn-659C	Lys-704C	Leu-597C			
		Asp-963C			Lys-603C	Met-689C	Cys-633C			
					Arg-604C	Leu-686C	Arg-601C			
					Trp-605C	Leu-707C	Leu-637C			
						Leu-708C	Val-596C			

		Binding site 1			Binding site 2			Binding site 3		
6PQQ-based model	score	8.34			5.21			4.52		
	Site	Channel gap			pre-S1 / S4 / S5 / TRP-like domain			pre-S1 / S4 / S5 / TRP-like domain		
		Val-942A	Pro-949D	Gly-914D	Val-875D	Arg-852B	Thr-874D	Arg-1011C	Phe-1024A	His-719A
		Leu-881C	Asn-954D	Ile-916B	Phe-853B	Gln-979B	Cys-1025B	Cys-1021A	Leu-708A	Phe-716A
		Ile-905C	Ile-957D	Asn-917B	Leu-1023B	Leu-707B	Phe-1020B	Pro-1010A	Leu-712A	Met-720A
		Phe-909C	Leu-913B	Tyr-918B	Thr-1026B	His-983B	Cys-1021B	Phe-1017A	Glu-854A	Leu-723A
		Ile-906C	Pro-949B	Asn-917C	Leu-850B	Leu-708B	Phe-1017B	His-1018A	Arg-852A	Gly-715A
		Ile-878C	Met-953B	Tyr-918C	Phe-879D	Arg-975B	Arg-1011B	Val-1005A	Phe-853A	Leu-982A
		Leu-941A	Asn-954B	Asn-917A	Phe-846B	Glu-1028B	Leu-712B	Tyr-1006A	Gln-979A	Arg-975A
		Ile-916A	Ile-957B	Ser-910D	Leu-723B	Ile-1029B	Leu-698B	Pro-1007A	Asn-855A	Met-978A
		Ser-910C	Leu-902C	Ser-910A	Cys-856B	Gly-1027B	Cys-703B	Phe-1022A	Glu-1028A	Ile-858A
		Leu-913C	Val-935A	Arg-919C	Phe-716B	Lys-704B	Asn-699B	Cys-1025A	Cys-856A	Lys-974A
		Gly-914A	Tyr-926A	Glu-920C	Met-720B	Glu-1032B	Ile-1019B	Phe-800B	Leu-707A	Gln-851A
		Asp-915C	Pro-934A	Asn-917D	Asn-855B	Ile-976B	His-1018B	Met-801B	His-983A	Glu-981A
		Gly-914C	Leu-903C	Glu-920D	Phe-1024B	Glu-705B	Phe-1022B	Thr-1026A	Lys-704A	Ser-985A
		Phe-938A	Ile-946A	Ile-950C	Glu-854B	Leu-871D	Pro-1010B	Trp-711A	Glu-705A	Leu-848A
		Tyr-918A	Leu-867C	Ser-910B	Trp-711B	Arg-872D		Phe-1020A	Ile-976A	Tyr-849A
		Ala-939A	Leu-870C	Ile-916D						
		Leu-913A	Leu-871C	Tyr-918D						
		Asp-915A	Thr-874C	Phe-909B						
		Pro-949A	Ile-950A	Ile-950D						
		Met-953C	Phe-877C	Glu-924C						
		Pro-949C	Val-951A	Phe-909D						
		Met-953A	Met-912C	Thr-945B						
		Asn-954C	Leu-956C	Phe-909A						
		Ile-957C	Asp-915D	Leu-941B						
		Asn-954A	Ile-916C	Ile-906A						
		Ile-957A	Gly-914B	Val-942B						
		Leu-913D	Asp-915B	Ser-900C						
		Met-953D								

Table S4: Castp3.0 TRPA1 binding site prediction results

		Ankyrin repeats		Channel gap		Transmembrane region		TRP-like domain area	
3J9P-based model	number of sites	8		3		3		4	
		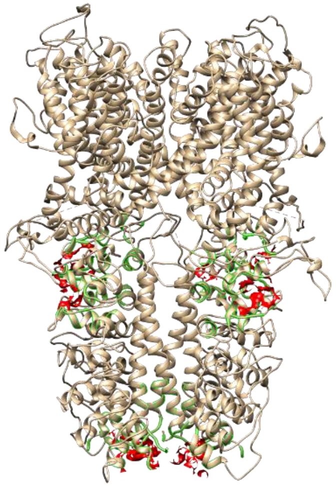		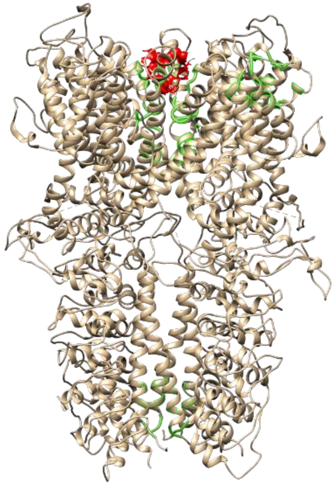		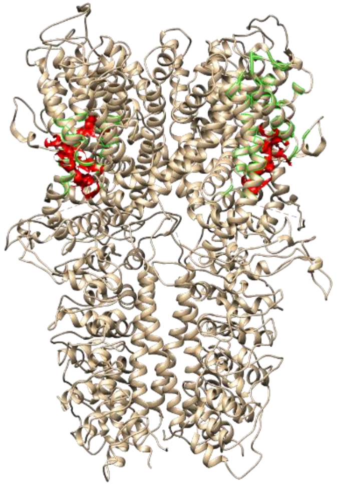		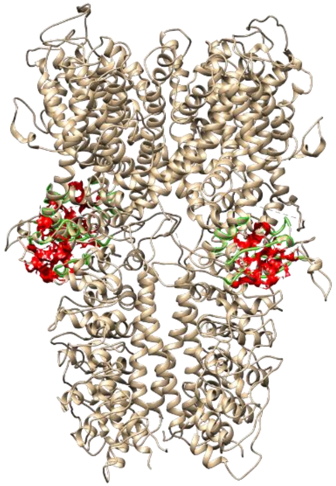	
	Sites' details	Area(SA)	Volume(SA)	Area(SA)	Volume(SA)	Area(SA)	Volume(SA)	Area(SA)	Volume(SA)
		376.322<br>379.370<br>362.644<br>345.352<br>234.976<br>234.714<br>234.632<br>143.230	119.040<br>118.614<br>112.002<br>108.928<br>96.300<br>96.083<br>96.028<br>63.240	661.154<br>225.302<br>177.676	521.175<br>79.644<br>56.523	234.741<br>225.302<br>165.347	96.208<br>79.644<br>77.629	262.030<br>252.168<br>241.639<br>177.457	192.827<br>169.087<br>131.245<br>123.297

		Ankyrin repeats		Channel gap		Transmembrane region		TRP-like domain area
6PQQ-based model	number of sites	5		2		11		
		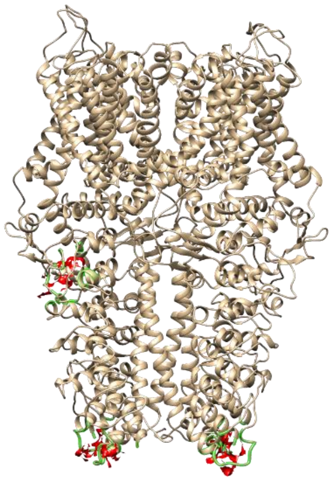		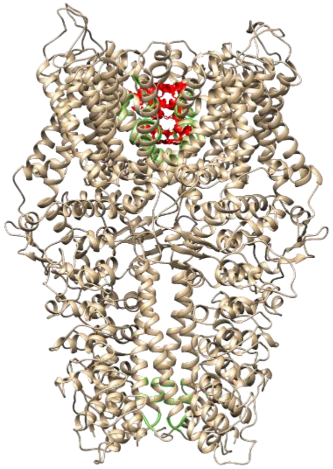		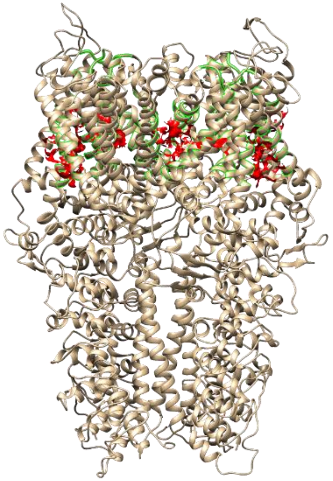		
	Sites' details	Area(SA)	Volume(SA)	Area(SA)	Volume(SA)	Area(SA)	Volume(SA)	
		171.229<br>123.463<br>79.200<br>74.575<br>118.121	87.070<br>61.535<br>64.834<br>52.827<br>50.780	602.144<br>155.912	556.536<br>81.334	157.806<br>155.348<br>150.966<br>139.597<br>78.232<br>102.544<br>110.502<br>97.413<br>53.778<br>90.840	89.004<br>79.688<br>74.437<br>64.020<br>54.091<br>36.835<br>36.183<br>33.812<br>31.333<br>31.313	

Table S5: List of active ligands used in retrospective virtual screening.

Ligand	Structure	SMILES	Patent	TRPA1 IC50
1	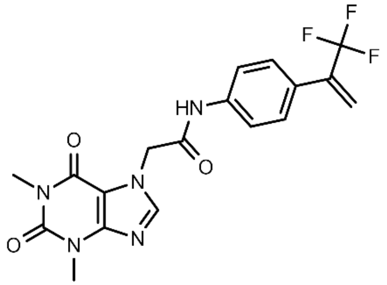	CN1C2=C(N(CC(=O)NC3=CC=C(C=C3)C(=C)C(F)(F)F)C=N2)C(=O)N(C)C1=O	WO2010138879	< 250 nM
2	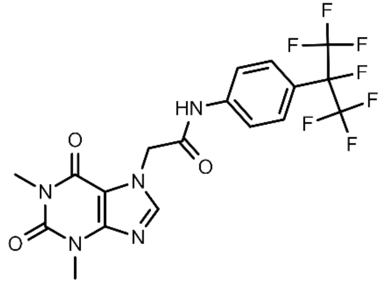	CN1C2=C(N(CC(=O)NC3=CC=C(C=C3)C(F)(C(F)(F)F)C(F)(F)F)C=N2)C(=O)N(C)C1=O	WO2010138879	<250 nM
3	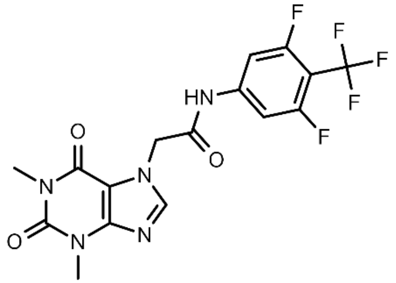	CN1C2=C(N(CC(=O)NC3=CC(F)=C(C(F)=C3)C(F)(F)F)C=N2)C(=O)N(C)C1=O	WO2010138879	250-499 nM

4	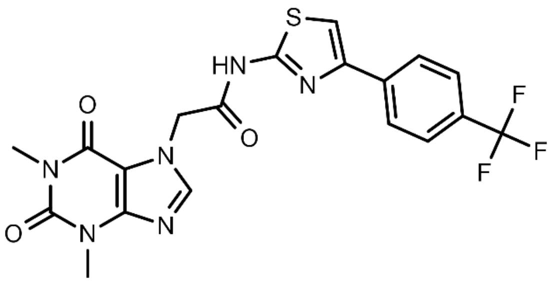	CN1C2=C(N(CC(=O)NC3=NC(=CS3)C3=CC=C<br>(C=C3)C(F)(F)F)C=N2)C(=O)N(C)C1=O	WO2009140519	<1 μM
5	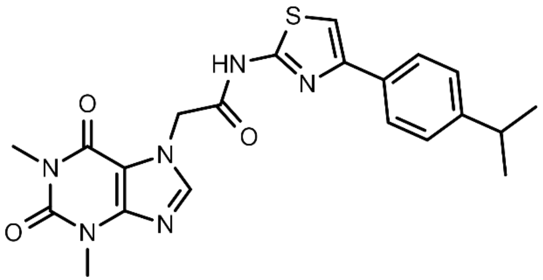	CC(C)C1=CC=C(C=C1)C1=CSC(NC(=O)CN<br>2C=NC3=C2C(=O)N(C)C(=O)N3C)=N1	WO2009002933	<1 μM
6	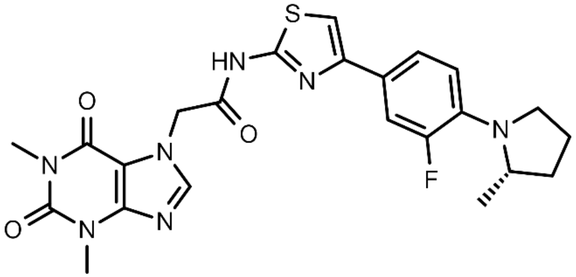	C[C@@H]1CCCN1C1=CC=C(C=C1F)C1=CSC(NC<br>(=O)CN2C=NC3=C2C(=O)N(C)C(=O)N3C)=N1	WO2010075353	4 nM
7	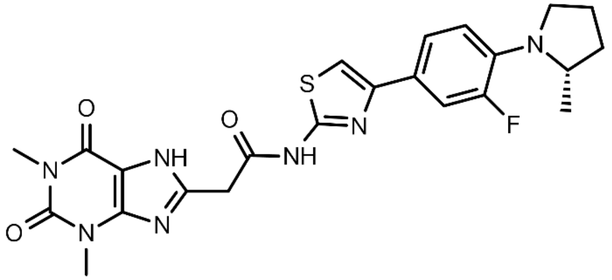	C[C@@H]1CCCN1C1=CC=C(C=C1F)C1=CSC(NC<br>(=O)CC2=NC3=C(N2)C(=O)N(C)C(=O)N3C)=N1	WO2010132838	310 nM

8	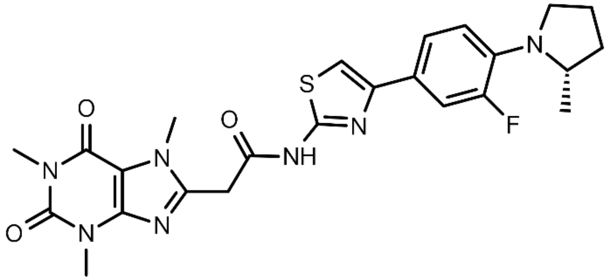	C[C@@H]1CCCN1C1=CC=C(C=C1F)C1=CSC(NC (=O)CC2=NC3=C(N2C)C(=O)N(C)C(=O)N3C)=N1	WO2010132838	81 nM
9	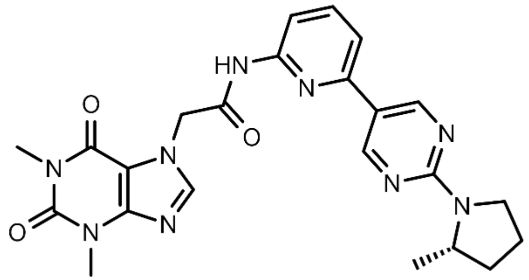	C[C@@H]1CCCN1C1=NC=C(C=N1)C1=CC=CC(NC (=O)CN2C=NC3=C2C(=O)N(C)C(=O)N3C)=N1	WO2013023102	93 nM
10	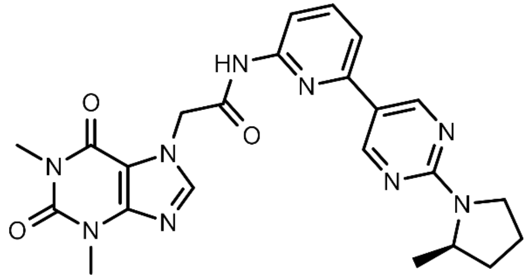	C[C@H]1CCCN1C1=NC=C(C=N1)C1=CC=CC(NC (=O)CN2C=NC3=C2C(=O)N(C)C(=O)N3C)=N1	WO2013023102	77 nM
11	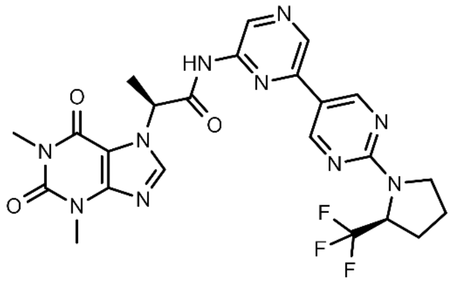	C[C@H](N1C=NC2=C1C(=O)N(C)C(=O)N2C)C(=O)NC1=NC (=CN=C1)C1=CN=C(N=C1)N1CCC[C@H]1C(F)(F)F	WO2014113671	< 25 nM

12	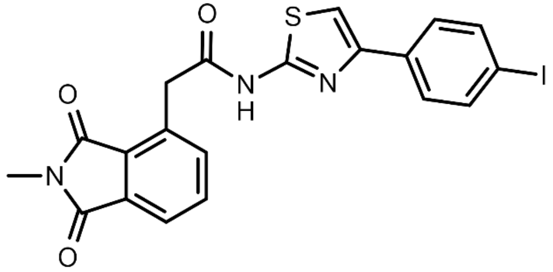	CN1C(=O)C2=CC=CC(CC(=O)NC3=NC<br>(=CS3)C3=CC=C(I)C=C3)=C2C1=O	WO2009118596	< 250 nM
13	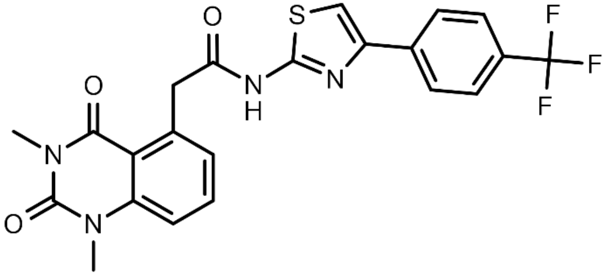	CN1C(=O)N(C)C2=CC=CC(CC(=O)NC3=NC<br>(=CS3)C3=CC=C(C=C3)C(F)(F)F)=C2C1=O	US20090325987	< 50 nM
14	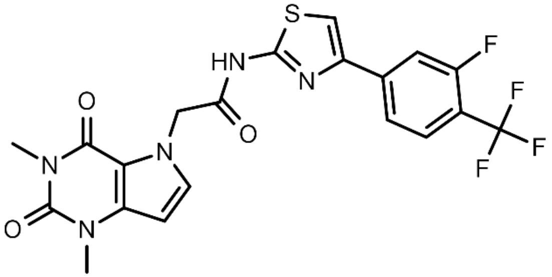	CN1C2=C(N(CC(=O)NC3=NC(=CS3)C3=CC=C<br>(C(F)=C3)C(F)(F)F)C=C2)C(=O)N(C)C1=O	WO2010109287	< 50 nM
15	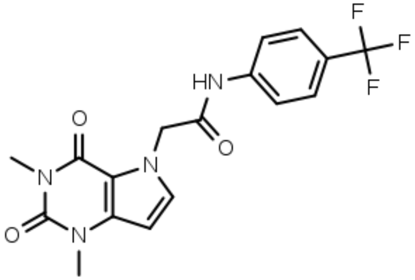	CN1C2=C(N(CC(=O)NC3=CC=C(C=C3)<br>C(F)(F)F)C=C2)C(=O)N(C)C1=O	-	400 nM

16	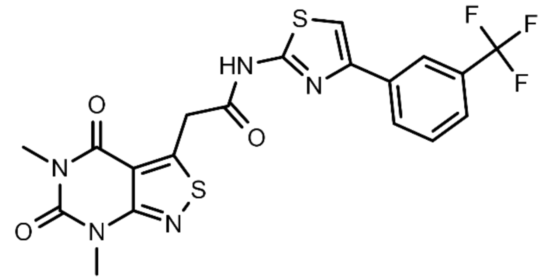	CN1C2=NSC(CC(=O)NC3=NC(=CS3)C3=CC=CC(=C3)C(F)(F)F)=C2C(=O)N(C)C1=O	WO2010109328	< 50 nM
17	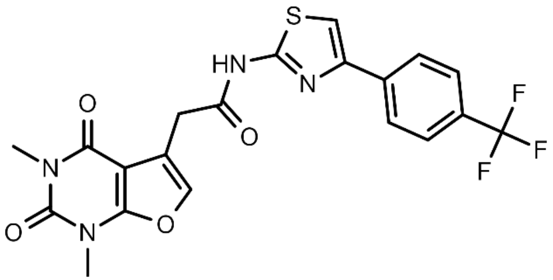	CN1C2=C(C(CC(=O)NC3=NC(=CS3)C3=CC=C(C=C3)C(F)(F)F)=CO2)C(=O)N(C)C1=O	WO2010109287	< 50 nM
18	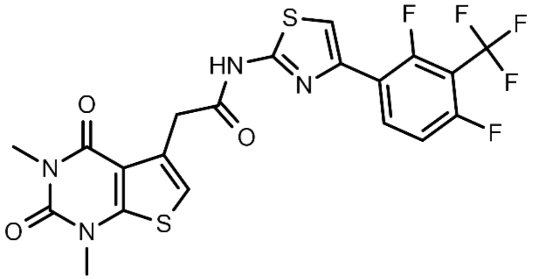	CN1C2=C(C(CC(=O)NC3=NC(=CS3)C3=CC=C(F)C(=C3F)C(F)(F)F)=CS2)C(=O)N(C)C1=O	WO2010109334	< 50 nM
19	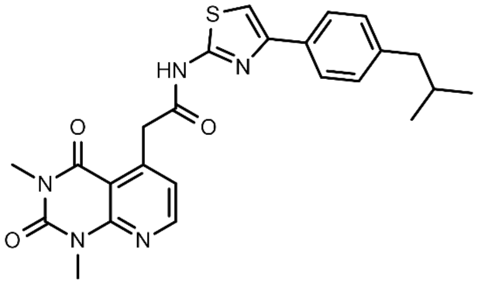	CC(C)CC1=CC=C(C=C1)C1=CSC(NC(=O)CC2=C3C(=O)N(C)C(=O)N(C)C3=NC=C2)=N1	WO2010125469	< 50 nM

20	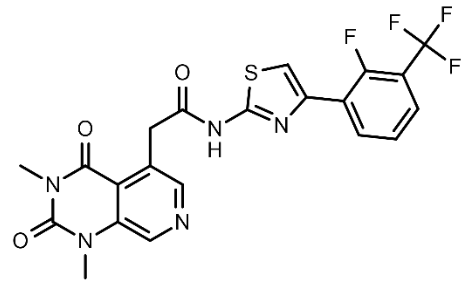	CN1CN(C)C2=CN=CC(CC(=O)NC3=NC(=CS3)C3=CC=CC(=C3F)C(F)(F)F)=C2C1=O	WO2011132017	< 50 nM
21	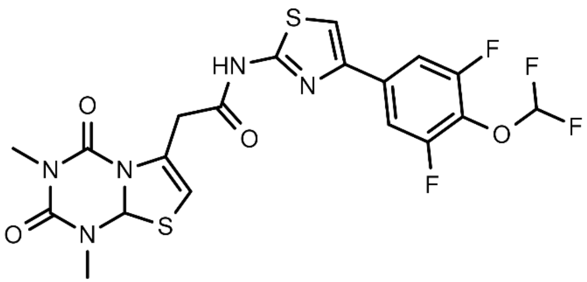	CN1C2SC=C(CC(=O)NC3=NC(=CS3)C3=CC(F)=C(OC(F)F)C(F)=C3)N2C(=O)N(C)C1=O	WO2011114184	< 50 nM
22	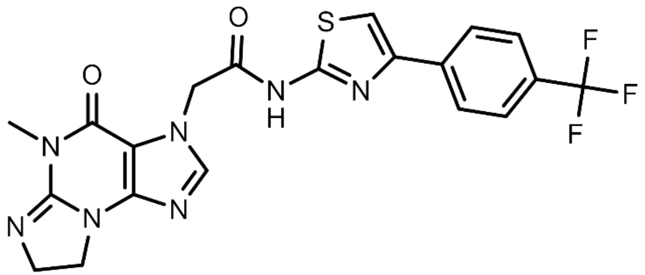	CN1C2=NCCN2C2=C(N(CC(=O)NC3=NC(=CS3)C3=CC=C(C=C3)C(F)(F)F)C=N2)C1=O	WO2009144548	< 500 nM
